# Editorial: Advances in immunity and microbiome: exploring key interactions and innovations

**DOI:** 10.3389/fimmu.2026.1933468

**Published:** 2026-09-03

**Authors:** Javier Pascual, Juan Fco Martínez-Blanch, Carmen Amaro, Francisco J. Roig

**Affiliations:** 1 Faculty of Health Sciences, Universidad Europea de Valencia, Valencia, Spain; 2 Archer Daniels Midland (ADM) Research and Development Center-Biopolis, Parc Científic Universitat de València, Paterna, Spain; 3 University Institute for Biotechnology and Biomedicine Research (BIOTECMED), Universitat de València, Burjassot, Spain; 4 Faculty of Health Sciences, Universidad San Jorge, Zaragoza, Spain; 5 BIAMICS: Servicios de Bioinformatica, Zaragoza, Spain

**Keywords:** microbiome, gut microbiota, immune modulation, microbiome-immune interactions, autoimmune diseases, inflammatory diseases, metagenomics and multi-omics, microbiome-based therapeutics

## Introduction

The relationship between the immune system and the microbiome is a rapidly evolving field that offers direct insight into the balance between health and disease. Advances in sequencing and omics technologies, including genomics, transcriptomics, epigenomics, and metagenomics, have deepened our understanding of how microbial metabolites influence immune cell function and how the microbiome shapes both innate and adaptive immunity, revealing therapeutic targets across autoimmune disease and infection. This Research Topic, entitled Advances in Immunity and Microbiome: Exploring Key Interactions and Innovations, collates 30 contributions from 210 authors, comprising original research, reviews, a systematic review and meta-analysis, a case report, and two correction notes. The Research Topic has already attracted early readership and citation across several specialties, reflecting broad interest in the field. It addresses four of the six themes proposed in the original call: mechanisms of microbiome-immune system interaction, the impact of gut microbiota on immune modulation, the role of the microbiome in inflammatory and autoimmune disease, and the influence of diet, lifestyle, and environment on immunity.

## Gut microbiota in autoimmune, joint, and bone disease

Rheumatoid arthritis (RA) is the most frequently addressed condition in this Research Topic. Zhan et al. review how traditional Chinese medicine formulations act on the gut-immune axis to modulate RA severity. Xie et al. examine the dual, context-dependent role of gut microbial metabolites, which act as both pathogenic and protective mediators in the same disease. A complementary mechanism is proposed by Guo et al. who trace how periodontopathic bacteria translocate signals from the oral cavity into RA pathogenesis, linking two mucosal compartments that are usually studied separately.

Beyond RA, Shen et al. report a systematic review and meta-analysis showing consistent depletion of the genus *Subdoligranulum* across multiple autoimmune diseases, which nominates it as a candidate cross-disease biomarker. Zhang et al. link intestinal microecological shifts to hyperuricemia and gouty arthritis. In a separate contribution, Xie et al. review the context-dependent, at times protective and at times destructive, role of the gut microbiota in bone remodeling and osteoporosis. Together, these studies place intestinal dysbiosis directly upstream of skeletal immune dysfunction.

## The oral-gut-systemic axis

Two contributions focus specifically on the mouth as an immunological entry point. Antequera et al. report associations between salivary lactoferrin, *Porphyromonas gingivalis* abundance, and stress hormone levels in periodontitis patients. A broader narrative review by the oral-gut microbiome group synthesizes the bidirectional mechanisms connecting oral dysbiosis to gastrointestinal and systemic inflammation. These findings confirm that the oral cavity is not a locally confined site of disease but an active node in systemic immune regulation.

## Microbiome interactions in reproductive, endocrine, and age-related physiology

Zheng et al. link dietary patterns to gut microbiome composition in gestational diabetes in a cohort of Chinese women, while Ma et al. implicate microbiome alterations in premature ovarian insufficiency. At the other end of the life course, Jayaraman et al. connect *Candida*-associated dysbiosis and fungal overgrowth to regulatory T cell dynamics during aging, and Vorontsov et al. review *Akkermansia muciniphila*, positioning this mucin-degrading commensal as a modulator of low-grade systemic inflammation, or inflammaging.

## Microbiome-immune crosstalk in oncology

The tumor microbiome is addressed at both the meta-analytic and mechanistic levels. Tang et al. map the expanding literature on microbial communities within tumor microenvironments, identifying emerging research clusters and collaboration networks. Qiu and Li use multi-omics integration to resolve crosstalk between the intratumoral microbiome, lactic acid metabolism, and immune status in lung squamous cell carcinoma. Two further studies extend this framework to specific organs: cervical cancer in a Mexican cohort (Klimov-Kravtchenko et al.) and prostate cancer, reviewed by Pei et al. These results confirm that microbiome-immune interactions are not restricted to the gastrointestinal tract.

## Infection, host defense, and experimental models

A substantial subset of this Research Topic advances mechanistic and translational understanding of host-pathogen interactions. In aquaculture, Kumar et al. characterize multidrug-resistant *Proteus penneri* in *Labeo rohita*, identifying an emerging veterinary and zoonotic concern. In murine models, Fu et al. describe inflammation driven by a new *Vibrio cholerae* O1 lineage in neonatal mice, and Chen et al. show that segmented filamentous bacteria (SFB) flagellin mediates epithelial adhesion, endocytosis, and immune regulation in germ-free mice. This experimental toolkit is extended by Garcia-Gonzalez et al., who generate a Paneth cell–specific Cre-recombinase transgenic mouse line, subsequently amended in a published correction (Garcia-Gonzalez et al.), providing a durable resource for dissecting Paneth cell biology in future gut-immune studies. At the clinical level, Tang et al. document dual-site pulmonary and peritoneal *Corynebacterium striatum* infection, characterized through genomic and immunologic profiling. Yang et al. link gut microbiota composition to inflammatory severity in community-acquired pneumonia, extending the gut-lung axis to a common respiratory infection, and do Nascimento et al. address humoral immunity to a major recent pathogen by profiling severity-dependent IgG epitope recognition in COVID-19.

## Microbial metabolites and systemic immune modulation

A final group of studies addresses how microbial metabolites and their cellular targets shape immunity beyond the gut wall. Xu et al. review short-chain fatty acids as key antiviral mediators; this is the most-viewed and most-cited article in this Research Topic. Liu et al. examine butyrate specifically for its immunomodulatory roles in asthma, and, in a separate review, Liu et al. analyze the crosstalk between probiotic strains and T cell responses. Ma et al. discuss pentraxin-3 in the context of lung infections. Pentraxin-3 is an acute-phase innate immune protein. Its expression is not constitutive. It is induced in macrophages, dendritic cells, neutrophils, and epithelial cells when Toll-like receptors and other pattern-recognition receptors engage microbial components, and when pro-inflammatory cytokines such as IL-1β and TNF-α accumulate during infection. Microbial exposure therefore shapes pentraxin-3 levels by driving this receptor-dependent induction, which is why circulating pentraxin-3 rises with microbial and inflammatory burden in lung infection. Two further studies address less conventional intersections of behavior, microbiome, and immunity: Li et al. report gut microbial mediation of immune profiles among HIV-negative men who have sex with men, assessed by multi-omics, and Li et al. review gut microbiota alterations in irritable bowel syndrome, the second most-viewed article in the Research Topic, consistent with sustained clinical interest in functional gastrointestinal disorders.

## Conclusions and future directions

Taken together, these 30 contributions demonstrate that microbiome-immune interactions are not confined to the gastrointestinal tract but extend to the oral cavity, respiratory system, reproductive organs, and tumor microenvironments. Across sections, three themes emerge consistently: the effects of specific taxa and metabolites are highly context-dependent, shifting between protective and pathogenic roles according to host status; multi-omics integration is increasingly required to move from association to mechanism; and a defined set of taxa and metabolites, including *Akkermansia muciniphila*, *Subdoligranulum*, short-chain fatty acids, and butyrate, repeatedly appear as candidate biomarkers or intervention targets across otherwise unrelated diseases.

Two directions warrant particular attention in future work. First, innovative *in vivo*, *in vitro*, and in silico models for studying microbiome-immunity interactions beyond conventional murine and cohort designs are needed. Second, therapeutic strategies targeting the microbiome-immune axis require direct evaluation in clinical and interventional settings, since the present contributions approach this question through mechanistic review rather than through trials of intervention. The strong early engagement with this Research Topic indicates that microbiome-immune crosstalk is now regarded as a mainstream determinant of disease across specialties as diverse as rheumatology, oncology, reproductive medicine, and infectious disease. Bridging the gap between mechanistic characterization and therapeutic testing, together with replication of the predominantly exploratory associations reported here in larger, prospective cohorts, defines the trajectory this field is poised to follow.

